# *Sedum mexicanum* Britt. Induces Apoptosis of Primary Rat Activated Hepatic Stellate Cells

**DOI:** 10.1155/2015/194373

**Published:** 2015-05-20

**Authors:** Shou-Lun Lee, Ting-Yu Chin, Ching-Long Lai, Wen-Han Wang

**Affiliations:** ^1^Department of Biological Science and Technology, College of Life Sciences, China Medical University, 91 Hsueh-Shih Road, Taichung 40402, Taiwan; ^2^Department of Bioscience Technology, Chung Yuan Christian University, 200 Chung Pei Road, Taoyuan 32023, Taiwan; ^3^Department of Nursing, Chang Gung University of Science and Technology, 261 Wenhwa 1st Road, Taoyuan 33303, Taiwan; ^4^Department of Biotechnology, Asia University, 500 Lioufeng Road, Taichung 41354, Taiwan

## Abstract

*Background*. Liver fibrosis is a significant liver disease in Asian countries.* Sedum mexicanum* Britt. (SM) has been claimed to have antihepatitis efficacy. In traditional folk medicine, a solution of boiling water-extracted SM (SME) is consumed to prevent and treat hepatitis. However, its efficacy has not yet been verified. The purpose of this study was to investigate the in vitro effect of SME on hepatoprotection. *Methods*. Hepatic stellate cells (HSCs) and hepatocytes (HCs) were isolated from the livers of the rats by enzymatic digestion and density gradient centrifugation. *Results*. Treating the HCs and aHSCs with SME caused a dose-dependent decrease in the viability of aHSCs but not that of HCs. In addition, treatment with SME resulted in apoptosis of aHSCs, as determined by DAPI analysis and flow cytometry. SME also increased the amount of cleaved caspase-3, cleaved caspase-9, and cleaved poly ADP-ribose polymerase (PARP) in aHSCs. Furthermore, SME treatment induced a dose-dependent reduction in Bcl-2 expression and increased the expression of Bax in aHSCs. *Conclusions*. SME did not cause cytotoxicity in HCs, but it induced apoptosis in aHSCs through the mitochondria-dependent caspase-3 pathway. Therefore, SME may possess therapeutic potential for liver fibrosis.

## 1. Introduction

Epidemiological studies have identified a number of factors, such as ethanol, viral infections, and metabolic diseases, which contribute to the risk of developing liver cirrhosis [1]. Alcoholic liver disease, hepatitis C, and nonalcoholic fatty liver disease are the most common causes of cirrhosis in developed countries [2], whereas hepatitis B is the prevailing cause of cirrhosis in the Asia-Pacific region [3, 4]. A report from the World Health Organization (WHO) indicated that an estimated two billion and 150 million people have been infected with the hepatitis B virus (HBV) and the hepatitis C virus (HCV), respectively. The worldwide burden of disease due to acute hepatitis B and hepatitis C infection and due to cancer and cirrhosis of the liver is high (approximately 2.7% of all deaths) (http://www.who.int/immunization/topics/hepatitis/en/). Accordingly, the treatment and prevention of liver cirrhosis are one of the most important issues clinically.

Liver cirrhosis is the end-stage of the pathology of various chronic liver diseases, and liver fibrosis is the precursor of liver cirrhosis [5]. The pathogenesis of liver fibrosis is regulated by the various types of myofibroblasts, which are the primary collagen-producing cells [6]. Most extracellular matrix (ECM) components are produced by activation of hepatic stellate cells (HSCs; also termed retinoid storing cells, fat-storing cells, and Ito cells). At the time of hepatic injury, quiescent HSCs transform to a myofibroblast-like phenotype, that is, activated HSCs (aHSCs), with contractile, proinflammatory, and potent fibrogenic activities [7]. The activation of HSCs is a critical step in the development of liver fibrosis and the inhibition of hepatocyte regeneration; however, reduced hepatocyte regeneration is a characteristic of liver disease and is related to fibrogenesis [8]. Therefore, modulation of decreased HSC activation and increased levels of HSC apoptosis could be an important complementary pathway in the pathogenesis of liver fibrosis [9]. Freshly isolated HSCs cultured on plastic dishes can be spontaneously activated in a manner similar to HSC activation in vivo; thus, this is a good model for liver fibrosis research [10].

Reports suggest that the reversal of liver fibrosis in patients is possible [11, 12]. Nowadays, traditional Chinese herbal medicine uses herbs to treat chronic liver disease and cirrhosis. Studies have shown that several compounds or extracts from Chinese herbal medicines, such as* Salvia miltiorrhiza*, are effective in the treatment of liver fibrosis and cirrhosis [13]. Different signaling pathways may be involved in aHSCs apoptosis. Chor et al. [14] reported that the extracts of several herbs, including Angelica sinensis, Carthamus tinctorius, Ligusticum chuanxiong, Salvia miltiorrhiza and Stephania tetrandra, can induce the apoptosis of aHSCs by upregulation of Fas and Bax and downregulation of Bcl-xL in aHSCs.* Sedum mexicanum* Britt. (SM) is mainly distributed in Taiwan north mountain [15]. SM has been proposed to possess antihepatitis efficacy, but this has not been proven. Furthermore, in traditional folk medicine, a beverage of SM boiled with hot water is consumed to prevent and treat hepatitis. However, the hepatoprotective effect of SM is poorly understood. Therefore, this study was designed to investigate the effects of the hot water extract of SM (SME) on aHSCs and hepatocytes (HCs). We found that the extract was able to induce apoptosis in aHSCs but not in HCs.

## 2. Methods

### 2.1. Chemicals and Reagents

Anti-*β*-actin antibodies, 4,6-diamidino-2-phenylindole dihydrochloride (DAPI), dexamethasone (DEX), formaldehyde, insulin, HEPES, Nycodenz, propidium iodide (PI), and Triton X-100 were purchased from Sigma-Aldrich (Missouri, USA). Fetal bovine serum (FBS) was purchased from Thermo Fisher Scientific Inc. (NY, USA). Percoll and pronase were purchased from GE Healthcare Bio-Sciences AB (Uppsala, Sweden). Trypan blue solution (0.5%) was purchased from BioWest (Nuaille, France). All other reagents were purchased from Invitrogen (CA, USA).

### 2.2. Preparation of SME

The hot water extract of* Sedum mexicanum* Britt. used in this study was obtained by adding freshly cleaned leaves and stems (20 grams) to 100 mL of boiling H_2_O for 1 h. Then, the* Sedum mexicanum* extract (SME) was filtered, lyophilized, and stored at −20°C. The powdered SME was dissolved in water, and a stock solution was fresh prepared at concentration of 1 g/mL before cell culture experiments.

### 2.3. Hepatic Stellate Cell and Hepatocyte Isolation

Male Wistar rats (200–250 g) were purchased from BioLASCO Taiwan Co., Ltd. (Taipei, Taiwan) and were housed in an environmentally controlled animal facility at 22°C with a daily 12 h light-dark cycle. The rats had free access to regular chow and water ad libitum. The experimental procedures used in the present study were approved by the Institutional Animal Care and Use Committee of China Medical University. Hepatic stellate cells (HSCs) and hepatocytes (HCs) were isolated from the livers of the rats (250–300 g) by enzymatic digestion and density gradient centrifugation, as described previously [16, 17] with some modifications. Briefly, the rat liver was perfused through a portal vein catheter using Ca^2+^- and Mg^2+^-free Hanks' balanced salt solution (HBSS); the solution was then changed to HBSS containing 5 mM Ca^2+^, 0.02% collagenase IV, and 0.1% pronase. The liver was removed from the rat and was gently torn with forceps in HBSS containing 0.01% DNase I. The cell suspension was filtered through a sterile nylon mesh, following centrifugation at 50 ×g for 7 min at 4°C. After centrifugation, HSCs and HCs were distributed in the supernatant and pellet, respectively. HSC and HC purification was performed using Nycodenz and Percoll gradient centrifugation, respectively. The suspension of HSCs was loaded on top of 18% Nycodenz at a proportion of 1 : 2. After centrifugation at 1,400 ×g for 10 min at 4°C, the HSC-enriched fraction was located in the upper whitish layer. The cell viability was assayed using Trypan blue exclusion and was routinely over 90%. The cell purity was determined by vitamin A autofluorescence at 328 nm excitation under a fluorescent microscope and was more than 90%. Additionally, the suspension of HC was loaded on top of a 25/50% Percoll gradient at a proportion of 1 : 2 : 2 following centrifugation at 1,800 ×g for 15 min at 4°C; the HCs were located in the pellet. HC viability was greater than 90%, as determined by Trypan blue exclusion. The purity of the cells was determined by light microscopy and was over 95%.

### 2.4. Cell Culture

Primary rat HSCs were grown in Dulbecco's modified Eagle's medium (DMEM) with low glucose containing 10% (v/v) fetal bovine serum (FBS), 100 U/mL penicillin, and 100 *μ*g/mL streptomycin in plates (10^5^ cells/mL) at 37°C in a humidified atmosphere of 5% CO_2_. The medium was changed every three days. HSCs can undergo activation by culturing them on uncoated plastic plates, which transforms them from the quiescent state to the fibrogenic myofibroblast-like phenotype, that is, activated HSCs (aHSCs). aHSCs were identified by measuring the expression of *α*-smooth muscle actin, which is a marker of stellate cell activation, and the cell purity was greater than 95%. aHSCs used for the experiments were at passages 1–3.

Primary HCs were suspended in William's Medium E containing 10% (v/v) FBS, 100 U/mL penicillin, 100 *μ*g/mL streptomycin, 2 mM L-glutamine, 0.86 *μ*M insulin, 0.5 nM dexamethasone, and 10 mM HEPES and were plated on collagen-coated dishes (5 × 10^5^ cells/mL). Cells were cultured at 37°C with 5% CO_2_ for 3 h for attachment and were washed twice with PBS. The medium was then changed, and, after overnight incubation, the cells were used for experiments.

### 2.5. Cell Proliferation and Viability

aHSCs and HCs were treated with different concentrations of SME for the indicated times. Cell proliferation was determined by MTT metabolic analysis. MTT was added to the cell medium, and, after incubation at 37°C for 4 h, the blue formazan reduction product was dissolved in isopropanol and measured on an ELISA reader at 570 nm. Viable cells were counted using a hemocytometer under a microscope after the cells were stained with Trypan blue.

### 2.6. DAPI Staining

aHSCs were cultured in 6-well plates and treated with or without SME for 72 h. The cells were washed with PBS, fixed with 4% formaldehyde for 10 min, and washed repeatedly with PBS. The cells in each well were stained with DAPI for 15 min before fixation with 0.1% Triton X-100 for 15 min. The chromatin changes were observed under a fluorescent microscope.

### 2.7. Apoptosis Analysis

aHSCs were incubated with or without SME in 6-well plates for 72 h. Apoptosis was determined using annexin V/PI double staining with an Annexin V-FITC Apoptosis Detection kit (BD Biosciences Pharmingen, CA). The cells were analyzed by flow cytometry using the Becton-Dickinson FACSCanto and BD CellQuest Pro software programs.

### 2.8. Western Blot Analysis

aHSCs were treated as indicated, detached, thoroughly washed with PBS, and then lysed in ice-cold lysis buffer. Following centrifugation at 13,000 ×g for 10 min at 4°C, the supernatants (30 *μ*g protein) were boiled with reducing sample buffer for 5 min, subjected to electrophoresis in SDS-polyacrylamide gels, and then transferred onto a PVDF membrane. The membrane was blocked with 1% BSA in PBS containing 0.1% Tween-20 (PBST) for 1 h at room temperature and then washed with PBST. Proteins were detected by incubating the membrane overnight at 4°C with antibodies against *α*-SMA, *β*-actin (Sigma-Aldrich, MO), Bak, Bal-2 (C21), Bax, caspase-3 (H-277), caspase-9 (H-170) (Santa Cruz Biotechnology, CA), cleaved caspase-9 (Asp 353), and cleaved PARP (Asp 214) (Cell Signaling Technology, MA). Next, the membrane was incubated with a primary antibody, and, finally, the membrane was incubated with a secondary antibody conjugated to horseradish peroxidase (HRP) for 1 h. An enhanced chemiluminescence (ECL) kit (Amersham Biosciences, IL, or Millipore, MA) was used for protein detection. The relative intensity of the immunoreactive bands was assessed using Image J software.

### 2.9. Statistical Analyses

The cultured primary HSCs and HCs isolated from rat liver were used in the study. The cultured cells were performed in duplicate for each experiment. Three independent experiments (*n* = 3; i.e., using cell preparations in duplicate from 3 rat livers) were carried out for any sets of experiments. Data are presented as means ± standard deviation (SD) for three independent experiments. The significant differences in the mean values were assessed using unpaired Student's *t*-test. Significance was defined as *p* < 0.05 (^*∗*^) and *p* < (^*∗∗*^) versus the appropriate control group.

## 3. Results

### 3.1. Effects of SME on Activated Hepatic Stellate Cell and Hepatocyte Survival

**Figure 1:**
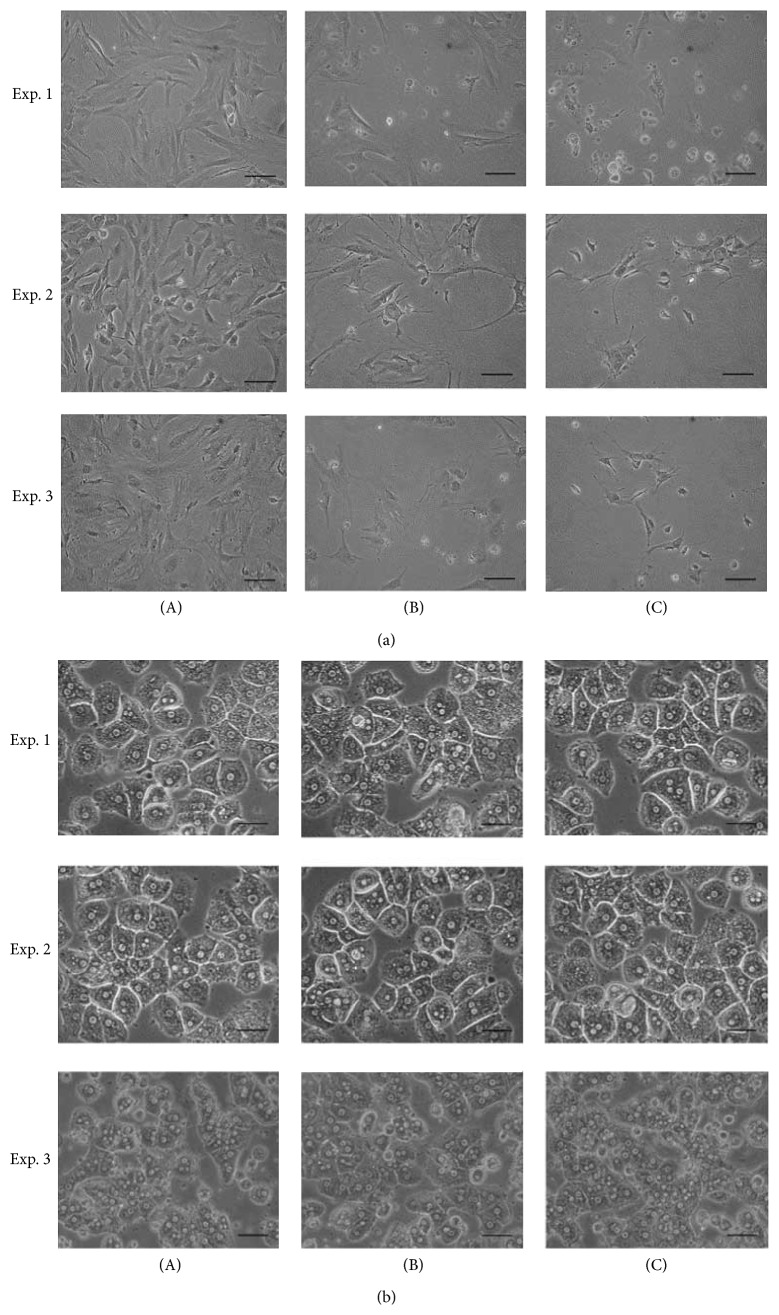
Effects of SME on aHSC and HC morphology. aHSCs (a) and HCs (b) were cocultured with SME at 0 (A), 0.3 (B), and 0.5 (C) mg/mL for 72 h and were observed under a microscope. Three independent experiments (*n* = 3; i.e., using cell preparations from 3 rat livers) were carried out for any sets of experiments. The scale bar equals 50 *μ*m.

**Figure 2:**
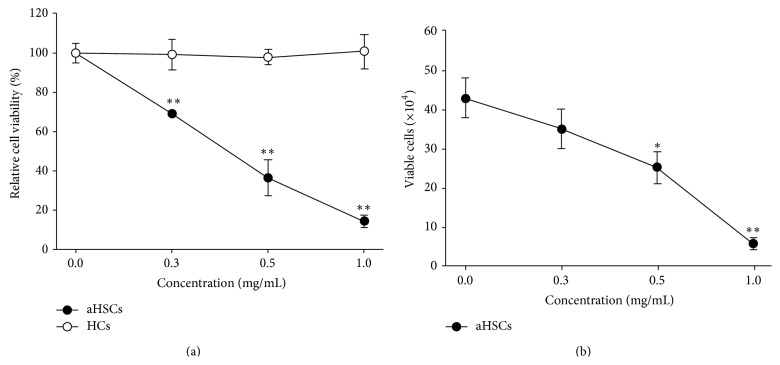
Effects of SME on aHSC and HC viability. aHSCs (●) and HCs (○) were treated with SME (0, 0.3, 0.5, and 1.0 mg/mL) for 72 h. Cell viability was determined by an MTT assay (a); the number of viable aHSCs was determined by a Trypan blue exclusion assay (b). The results are presented as the means ± SD. ^*∗*^ *p* < 0.05, ^*∗∗*^ *p* < 0.01, compared with control (0 mg/mL SME).

Activated hepatic stellate cells (aHSCs) and hepatocytes (HCs) were treated with several concentrations of SME for 72 h. Untreated aHSCs were homogenously distributed in the culture field, but SME-treatment resulted in cell shrinkage and cell debris in the culture medium (Figure 1(a)). However, the morphology of HCs was consistent regardless of whether the cells were treated with SME or not (Figure 1(b)). Additionally, the growth of aHSCs was significantly inhibited by SME in a dose-dependent manner, as determined by MTT assay (Figure 2(a)). However, the growth of HCs was not decreased compared to the cells cultured with various concentrations of SME (Figure 2(a)). Furthermore, the Trypan blue exclusion test indicated that SME reduced the number of aHSCs in a dose-dependent manner (Figure 2(b)). Therefore, the results demonstrated that SME possessed cytotoxic activity in aHSCs, but not in HCs.

### 3.2. SME-Induced Apoptosis of aHSCs

**Figure 3:**
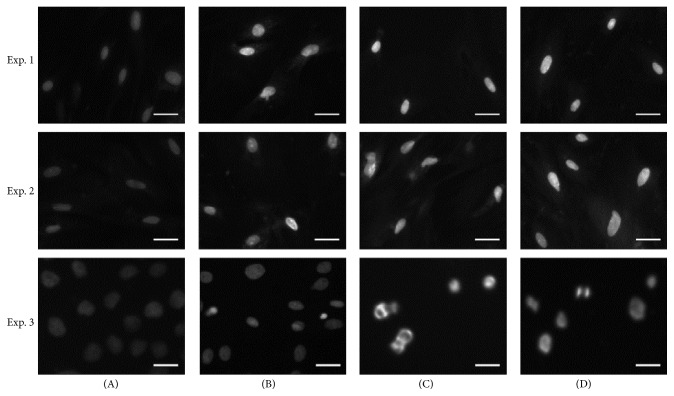
Determination of SME-induced apoptosis of aHSC. aHSCs were cultured with SME at 0 (A), 0.3 (B), 0.5 (C), and 1 (D) mg/mL for 72 h. Then, the cells were stained with DAPI and were observed under a fluorescence microscope. Three independent experiments (*n* = 3; i.e., using cell preparations from 3 rat livers) were carried out for any sets of experiments. The scale bar equals 50 *μ*m.

**Figure 4:**
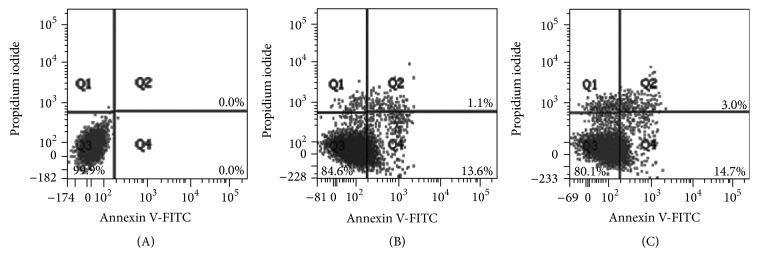
Flow cytometric analysis of aHSC exposed to SME. aHSCs were exposed to SME at concentrations of 0 (A), 0.3 (B), and 0.5 (C) mg/mL for 72 h. The cells were harvested and incubated with FITC-conjugated annexin V and PI and were measured by flow cytometry. Normal cells were annexin V- and PI-negative (Q3); cells in early apoptosis were annexin V-positive and PI-negative (Q4); cells in late apoptosis/necrosis were annexin V- and PI-positive (Q2).

**Figure 5:**
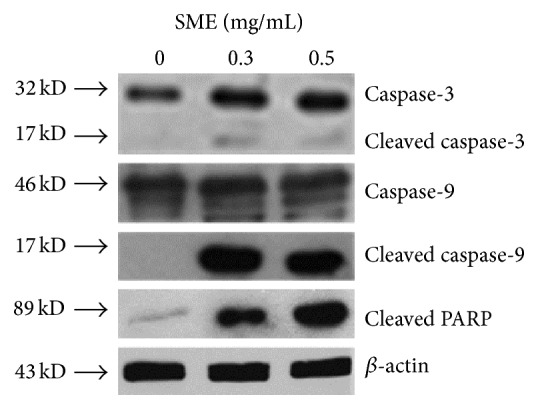
Effects of SME on the expression of apoptosis-associated proteins in aHSC. aHSCs were incubated with SME (0, 0.3, and 0.5 mg/mL) for 72 h. The cells were harvested and analyzed for caspase-3, caspase-9, cleaved caspase-9, cleaved PARP, and *β*-actin by western blot analysis.

Our findings show that SME had an inhibitory effect on aHSC viability. We further investigated whether the reduction in cell number by SME involved apoptosis. DAPI staining showed that chromatin condensation and nuclear shrinkage in aHSCs occurred after SME-treatment (Figure 3). In addition, annexin V/PI double staining indicated that SME induced an increase in the number of cells in both early apoptosis and late apoptosis/necrosis through apoptosis analysis by flow cytometry (Figure 4). These results showed that the number of cells in early apoptosis was 12.8–15.7% for the SME-treated cells and 0.1% for the untreated cells. Furthermore, the number of cells in late apoptosis/necrosis was 1.5–3.2% for the SME-treated cells and 0.1% for the untreated cells (Table 1). For the SME-treated aHSCs, apoptosis analysis by flow cytometry indicated that the number of cells in early apoptosis was significantly increased. Furthermore, we explored the molecules associated with SME-induced apoptosis. Therefore, we examined the protein expression of caspase-3, caspase-9, and PARP by western blot analysis. The cleaved forms of caspase-3, caspase-9, and PARP were increased in SME-treated aHSCs (Figure 5). Therefore, the results suggest that SME induced aHSC apoptosis.

### 3.3. Effects of SME on the Modulation of Bcl-2 Family Proteins

**Figure 6:**
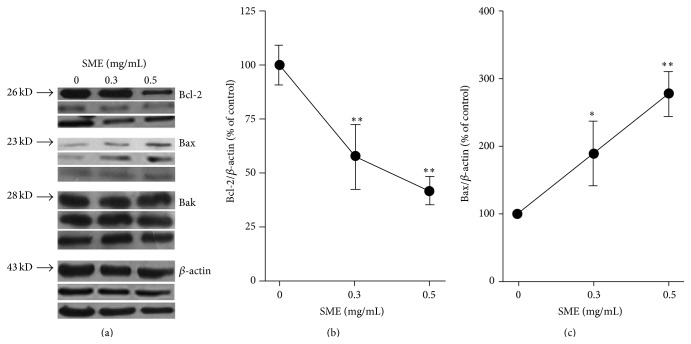
Effects of SME on the expression of Bcl-2 family proteins in aHSC. aHSCs were treated with the indicated concentrations of SME for 72 h. The expression levels of Bcl-2, Bax, Bak, and *β*-actin were measured by western blot analysis (a). Quantitative densitometric analysis was used to calculate the values of Bcl-2 (b) and Bax (c) normalized against *β*-actin. Three independent experiments (*n* = 3; i.e., using cell preparations from 3 rat livers) were carried out for any sets of experiments. The results are presented as the means ± SD. ^*∗*^ *p* < 0.05, ^*∗∗*^ *p* < 0.01, compared with control (0 mg/mL SME).

The mitochondria-mediated pathway is the major pathway of apoptotic cell death that is controlled by Bcl-2 family proteins [18]. We investigated whether the intrinsic mitochondrial apoptotic pathway was involved in SME-induced apoptosis. The antiapoptotic proteins (e.g., Bcl-2) and the proapoptotic proteins (e.g., Bax) of the Bcl-2 family are important for determining apoptosis [19]. The immunoblot analysis showed that SME significantly reduced the amount of Bcl-2 and increased the amount of Bax in aHSCs, but Bak did not significantly differ between the treatment and control groups (Figure 6(a)). We found that Bcl-2 was reduced in a dose-dependent manner with significant reductions of 42% and 58% for the 0.3 mg/mL and 0.5 mg/mL SME-treatments, respectively (Figure 6(b)). The expression of Bax was significantly increased by 88% and 176% for the 0.3 mg/mL and 0.5 mg/mL SME-treatments, respectively (Figure 6(c)). These results indicated that SME induced aHSC apoptosis through the mitochondria-dependent pathway.

## 4. Discussion

Hepatocyte apoptosis and hepatic stellate cell activation are major contributors to the fibrogenic process [20]. Hepatotoxic agents target HCs resulting in HC apoptosis and liver injury contributing to liver inflammation, fibrogenesis, and cirrhosis [11]. SME-treatment did not affect the morphology and viability of HCs (Figures 1 and 2), showing that SME-treatment was not cytotoxic to HCs. In addition, HSC activation increased proliferation and extracellular matrix accumulation, which is characteristic of hepatic fibrogenesis [21]. SME-treatment significantly affected the morphology and viability of aHSCs (Figures 1 and 2), indicating that SME-treatment was cytotoxic to aHSCs. A previous report indicated that reducing HC death and inducing HSC death may be a viable strategy for liver fibrosis therapy [5]. Therefore, SME has potential as a protective agent against liver fibrosis.

Recent studies have shown that selective stimulation apoptosis in aHSCs rather than HCs inhibits fibrosis [10]. Iredale and colleagues demonstrated that the spontaneous recovery of liver fibrosis was due to the apoptosis of HSCs [22]. Apoptosis can be induced through the extrinsic and intrinsic pathways; these pathways converge with the activation of caspase-3, which cleaves a number of substrates, for example, PARP [23]. Cleaved PARP is regarded as a marker for the execution phase of the apoptosis response [24]. SME-treatment induced aHSC apoptosis as determined by DAPI analysis and flow cytometry (Figures 3 and 4). SME also increased the amount of cleaved caspase-3 and cleaved PARP in aHSCs (Figure 5). Our data indicate that SME induced aHSCs death through a caspase-3-dependent apoptotic pathway; however, cell necrosis cannot be completely excluded (Table 1). Additionally, SME-treatment significantly increased the content of cleaved caspase-9 in aHSCs (Figure 5), which suggests that SME-induced apoptosis may occur through the intrinsic pathway. Activation of caspase-9 is induced by mitochondrial cytochrome c release and apoptosome assembly, which is modulated by Bcl-2 family proteins [18]. aHSCs treated with SME showed a dose-dependent reduction in Bcl-2 and an increase in the expression of Bax, which are antiapoptotic and proapoptotic proteins, respectively (Figure 6). These results suggest that SME induces aHSC apoptosis through the mitochondria-dependent pathway. However, the susceptibility of aHSCs to proapoptotic stimuli is different between rodent and human cells [25]. Human HSCs are resistant to apoptosis; thus, SME-induced apoptosis of human aHSCs can be investigated. Additionally, further in vivo studies of the efficacy of SME against liver fibrosis are necessary.

This is the first demonstration of the hepatoprotective effects of* Sedum mexicanum* Britt. Traditional Chinese herbal medicine has been used as conventional or complementary medicines for the treatment of liver fibrosis, such as* Stephania tetrandra* S. Moore and* Curcuma longa* L. [26]. The active compound that can induce the apoptosis of aHSCs extracted from* Stephania tetrandra* S. Moore and* Curcuma longa* L. is tetrandrine and curcumin, respectively [27, 28]. Chor et al. reported that five herbs, namely,* Angelica sinensis*,* Carthamus tinctorius*,* Ligusticum chuanxiong*,* Salvia miltiorrhiza*, and* Stephania tetrandra*, demonstrated both antiproliferative and proapoptotic activities in HSC-T6. These results were associated with upregulation of Fas and Bax and downregulation of Bcl-xL in HSC [14]. Herein, we speculate that SME include specific ligands which play a central role in instructive apoptosis. The specific ligands may induce upregulation of Fas and Bax and downregulation of Bcl-2 resulting in HSC-specific killing. Therefore, the bioactive compound of* Sedum mexicanum* Britt. that induces aHSC apoptosis requires further identification.

## 5. Conclusions

Liver fibrosis is a significant liver disease in Asian countries. In traditional folk medicine, various herbs, such as* Sedum mexicanum* Britt., are thought to have hepatoprotective effects, but this efficacy has not been scientifically verified yet. Our data demonstrated that SME possessed cytotoxicity in aHSCs but not in HCs. Our results indicate that SME induces apoptosis in aHSCs through the mitochondria-dependent pathway. Therefore, SME has therapeutic potential against hepatic fibrogenesis. However, further in vivo investigations into the efficacy of the hepatoprotective effect of SME are necessary.

## Figures and Tables

**Table 1:** Quantification of apoptosis in aHSCs after SME-treatment.

SME (mg/mL)	Normal cells^a^	Cells in the early apoptosis^b^	Cells in the late apoptosis/necrosis^c^
0	99.6 ± 0.3%	0.1 ± 0.1%	0.1 ± 0.1%
0.3	84.4 ± 4.3%^∗∗^	12.8 ± 3.7%^∗∗^	1.5 ± 0.5%^∗^
0.5	79.4 ± 1.3%^∗∗^	15.7 ± 1.1%^∗∗^	3.2 ± 0.2%^∗∗^

aHSCs were treated with indicated concentrations of SME for 72 h. Cells were harvested and incubated with FITC-conjugated annexin V and PI. The results show the percentage of cells in various stages that were measured by flow cytometry. The values are presented as the means ± SD. ^∗^ *p* < 0.05, ^∗∗^ *p* < 0.01, compared with 0 mg/mL.

^a^Normal cells were annexin V- and PI-negative.

^b^Cells in early apoptosis were annexin V-positive and PI-negative.

^c^Cells in late apoptosis/necrosis were annexin V- and PI-positive.

## References

[1] Schuppan D., Afdhal N. H. (2008). Liver cirrhosis. *The Lancet*.

[2] Naveau S., Perlemuter G., Balian A. (2005). Epidemiology and natural history of cirrhosis. *La Revue du Praticien*.

[3] Ganem D., Prince A. M. (2004). Hepatitis B virus infection-natural history and clinical consequences. *The New England Journal of Medicine*.

[4] Liaw Y.-F., Leung N., Kao J.-H. (2008). Asian-Pacific consensus statement on the management of chronic hepatitis B: a 2008 update. *Hepatology International*.

[5] Zhou W.-C., Zhang Q.-B., Qiao L. (2014). Pathogenesis of liver cirrhosis. *World Journal of Gastroenterology*.

[6] Wynn T. A., Barron L. (2010). Macrophages: master regulators of inflammation and fibrosis. *Seminars in Liver Disease*.

[7] Li D., Friedman S. L. (1999). Liver fibrogenesis and the role of hepatic stellate cells: new insights and prospects for therapy. *Journal of Gastroenterology and Hepatology*.

[8] Ebrahimkhani M. R., Oakley F., Murphy L. B. (2011). Stimulating healthy tissue regeneration by targeting the 5-HT_2B_ receptor in chronic liver disease. *Nature Medicine*.

[9] Gong W., Pecci A., Roth S., Lahme B., Beato M., Gressner A. M. (1998). Transformation-dependent susceptibility of rat hepatic stellate cells to apoptosis induced by soluble Fas ligand. *Hepatology*.

[10] Friedman S. L. (2008). Mechanisms of hepatic fibrogenesis. *Gastroenterology*.

[11] Bataller R., Brenner D. A. (2005). Liver fibrosis. *Journal of Clinical Investigation*.

[12] Friedman S. L., Bansal M. B. (2006). Reversal of hepatic fibrosis—fact or fantasy?. *Hepatology*.

[13] Feng Y., Cheung K.-F., Wang N., Liu P., Nagamatsu T., Tong Y. (2009). Chinese medicines as a resource for liver fibrosis treatment. *Chinese Medicine*.

[14] Chor S. Y., Hui A. Y., To K. F. (2005). Anti-proliferative and pro-apoptotic effects of herbal medicine on hepatic stellate cell. *Journal of Ethnopharmacology*.

[15] Tang W. S., Huang T. C., Huang T. C. (1993). Crassulaceae. *Flora of Taiwan. Volume 3*.

[16] Knook D. L., Seffelaar A. M., de Leeuw A. M. (1982). Fat-storing cells of the rat liver. their isolation and purification. *Experimental Cell Research*.

[17] Smedsrod B., Pertoft H. (1985). Preparation of pure hepatocytes and reticuloendothelial cells in high yield from a single rat liver by means of Percoll centrifugation and selective adherence. *Journal of Leukocyte Biology*.

[18] Dewson G., Kluck R. M. (2009). Mechanisms by which Bak and Bax permeabilise mitochondria during apoptosis. *Journal of Cell Science*.

[19] Budihardjo I., Oliver H., Lutter M., Luo X., Wang X. (1999). Biochemical pathways of caspase activation during apoptosis. *Annual Review of Cell and Developmental Biology*.

[20] Mallat A., Lodder J., Teixeira-Clerc F., Moreau R., Codogno P., Lotersztajn S. (2014). Autophagy: a multifaceted partner in liver fibrosis. *BioMed Research International*.

[21] Parsons C. J., Takashima M., Rippe R. A. (2007). Molecular mechanisms of hepatic fibrogenesis. *Journal of Gastroenterology and Hepatology*.

[22] Iredale J. P., Benyon R. C., Pickering J. (1998). Mechanisms of spontaneous resolution of rat liver fibrosis: hepatic stellate cell apoptosis and reduced hepatic expression of metalloproteinase inhibitors. *The Journal of Clinical Investigation*.

[23] Elmore S. (2007). Apoptosis: a review of programmed cell death. *Toxicologic Pathology*.

[24] Lazebnik Y. A., Kaufmann S. H., Desnoyers S., Poirier G. G., Earnshaw W. C. (1994). Cleavage of poly (ADP-ribose) polymerase by a proteinase with properties like ICE. *Nature*.

[25] Kawada N. (2006). Human hepatic stellate cells are resistant to apoptosis: implications for human fibrogenic liver disease. *Gut*.

[26] Luk J. M., Wang X., Liu P. (2007). Traditional Chinese herbal medicines for treatment of liver fibrosis and cancer: from laboratory discovery to clinical evaluation. *Liver International*.

[27] Zhao Y.-Z., Kim J.-Y., Park E.-J. (2004). Tetrandrine induces apoptosis in hepatic stellate cells. *Phytotherapy Research*.

[28] Zheng S., Chen A. (2004). Activation of PPAR*γ* is required for curcumin to induce apoptosis and to inhibit the expression of extracellular matrix genes in hepatic stellate cells in vitro. *Biochemical Journal*.

